# The clinical utility of cystatin C based eGFR in assessing renal function among HIV/AIDs patients on ART at Mildmay Uganda

**DOI:** 10.1186/s12882-024-03581-3

**Published:** 2024-04-23

**Authors:** Enock Wekiya, Godfrey P. Mujuzi, Jane Nakiyingi, Juliet Sanya, Moses Matovu, Ocung Guido, Jane Nakaweesi, Charles Karamagi, Joan K. Nakayaga, Edrisa I. Mutebi, Damalie Nakanjako

**Affiliations:** 1 Mildmay Uganda, P.O Box 24985, Kampala, Uganda; 2 College of Health Sciences, Makerere University, P.O. Box 7072, Kampala, Uganda

**Keywords:** Cockcroft and Gault, Creatinine, Cystatin C based eGFR, Glomerular filtration rate, ROC analysis, Likelihood ratio, Specificity, Sensitivity

## Abstract

**Background:**

In clinical practice, Measurement of estimated glomerular filtration rates (eGFR) is the gold standard assessing renal function the glomerular filtration rate often estimated from plasma creatinine. Several studies have shown Cystatin C based eGFR (Cys C) to be a better parameter for the diagnosis of impaired renal function. Cystatin C based eGFR has been proposed as a potential renal function marker but its use in HIV&AIDS patients has not been well evaluated.

**Methods:**

A cross sectional study was carried out on 914 HIV&AIDS patients on antiretroviral therapy (ART) attending Mildmay Uganda for care and treatment between January to March 2015. Serum Cystatin C based eGFR was measured using the particle enhanced immunoturbidimetric assay. Creatinine was analyzed using enzymatic Creatinine PAP method and creatinine clearance was calculated according to C&G.

**Results:**

The sensitivity of Cystatin C based eGFR was 15.1% (95% CI = 8.4, 24) with specificity 99.3% (95% CI = 98- 99.7). The positive and negative predictive values were 70.0% (95% CI 45.7–88.1) and 91.2% (95% CI 98.11–92.94) respectively. The positive likelihood ratio was 18.81 and negative likelihood ratio was 0.85. Cystatin C based eGFR had diagnostic accuracy of 90.7 and area under curve was 0.768.

**Conclusion:**

Cystatin C based eGFR exhibited a high specificity and a high positive likelihood ratio in diagnosis of kidney disease among HIV&AIDS patients. Cystatin C based eGFR can be used as a confirmatory test.

## Introduction

Primarily, the acquired immunodeficiency syndrome (AIDS) is caused by infection with the Human Immunodeficiency Virus type 1 (HIV-1) and type 2 (HIV-2). However, globally, the majority of cases can be attributed to HIV-1 [1, 2]. There was an estimated 35.3 million people living with HIV in 2012 [3] and an estimated 39.0 million people globally were living with HIV in 2022 worldwide [4]. Approximately 20% of all HIV positive people and 40% of incident infections are found in sub-Saharan Africa amongst the youths [5]. In Uganda, the HIV prevalence is at 7.3% (UNAIDS, 2012) accounting for an estimated 1.4 million people living with HIV&AIDS [6].

HIV infected individuals are at increased risk of developing renal complications compared to the general population where this increased risk could be from HIV infecting renal cells leading to conditions like HIV associated nephropathy, HIV immune complex disease [7, 8]. Renal dysfunction increases the risk of death by 2.5 fold in HIV&AIDS patients [9–13]. Acyclovir, pentamidine, aminoglycoside antibiotics, acyclovir, foscarnet, amphotericin, tenofovir, and adefovir are among the medications that are frequently used in HIV-related medical conditions. Additional renal complications may result from drug toxicities to highly active antiretroviral therapy (HAART). Tenofovir has been linked to an increased risk of renal complications among the medications included in HAART regimens [14]. If unattended to,there is rapid progression to a life threatening condition, end stage renal disease (ESRD) which is associated with high mortality and high patient care cost that resource limited settings can’t afford [14, 15].

The Global Burden of Disease (GBD) 2015 study also estimated that, in 2015, 1.2 million people died from kidney failure, an increase of 32% since 2005 [5]. With an estimated 2.3–7.1 million people with end-stage kidney disease died without access to chronic dialysis [16]. A recent systematic review estimates the current prevalence of chronic kidney disease (CKD) in sub-Saharan Africa (SSA) at 13.9% [17]. In Uganda, the prevalence of kidney disease is estimated at 14.8% [18]. It is important that kidney disease in identified early and managed appropriately. Diagnosis of Kidney dysfunction is done by estimation of glomerular filtration rate. The gold standard for estimating renal function is by GFR determined by infusion of inulin or another suitable marker. These techniques are time- consuming, labour- intensive, expensive and require administration of substances that make them incompatible with routine monitoring [19–21]. Thus, the measurement of endogenous substance is a common practice. Creatinine is the most commonly used filtration marker in clinical practice, but, as was described in the guideline titled ‘Use of serum creatinine concentration to assess level of kidney function’, its accuracy is significantly hampered by assay interference, unreliability of urine collection, and the confounding influences of diet, age, gender and muscle mass [22, 23]. To compensate for the inadequacies of the creatinine concentration as a GFR marker, there had been several successful attempts at constructing GFR prediction equations including additional parameters and these include the Cockcroft-Gault Eq. [24], the current used gold standard. However, because of the issues associated with creatinine, these formulae coherently over estimates GFR.

A number of low molecular weight serum proteins, including b2-microglobulin, retinol-binding protein and Cystatin C based eGFR, have been proposed as suitable alternative endogenous filtration markers [25–27]. Of these, Cystatin C based eGFR has received the most interest in the published literature. Cystatin C based eGFR a 13 kDa protease inhibitor belonging to the Cystatin superfamily is a promising molecule. Cystatin C based eGFR is encoded by housekeeping gene and is produced by all nucleated cells [26]. It is freely filtered at the glomerulus and not secreted by the tubules. Unlike creatinine it is not affected by gender, age and muscle mass [28, 29]. There is limited data regarding the use of Cystatin C based eGFR concentration in HIV & AIDS infected individuals on ART. The aim of this study was to assess the clinical utility of Cystatin C based eGFR in HIV infected individuals on ART.

## Methods

A cross sectional study was carried out January 2015 to March 2015 at Mildmay Uganda specialized HIV&AIDS clinic located at Lweza. The 1032 participants were selected using systematic random sampling, with each research assistant enrolling 18 participants per day. Given that Mildmay receives 150 patients daily, 54 participants were enrolled per day, with each interview lasting approximately 20 min. The Principal Investigator randomly selected a starting point among the first seven patients and then enrolled every seventh participant who met the inclusion criteria and consented to participate. We included HIV/AIDS patients aged 18 years and above on ART receiving care from Mildmay Uganda and who had given informed consent. Patients unable to provide valid and sufficient samples and with thyroid dysfunction were excluded. Measurement variables included: age, weight, and sex, height, treatment regime, clinical characteristics of the patient, serum creatinine and serum Cystatin C based eGFR concentrations.

### Analytical methods

Patient demographic data was collected by research assistants using a pretested questionnaire/data collection tool, The research volunteers later entered the certified data into EPI data v 3.1 for export to STATA v.12 for analysis, A vacutainer blood collection system was used to collect 4mLs of blood from the vein into a 4 mL plain tube, Blood was left to stand for 30 min so as to coagulate and later centrifuged for 5 min at 3000revolution per minute. Samples were analyzed using Cobas C311 analyzer (Roche/Hitachi Cobas c systems, Mannheim, Germany). Serum Cystatin C based eGFR was measured by a quantitative sandwich enzyme immunoassay using the particle enhanced immunoturbidimetric assay. Creatinine was analyzed using enzymatic Creatinine PAP method [30]. The equipment was calibrated daily and normal and pathological controls were included in each run. Control charts were examined using Westgard rules before results were reported.

### Statistical methods

Data analysis was done using STATA version 12. Categorical variables were summarized as proportions, while means (SD), median (IQR) were used to summarize continuous variables that were normally distributed and not normally distributed respectively. Normal probability plots were used to check normality of the data. To compare the ability of Cystatin C based eGFR to predict renal dysfunction, study subjects were divided into those with normal kidney function using a cut-off point of GFR 60 mL/min/1.73m^2^ using the Cockcroft – Gault formula based on creatinine as gold standard, the same cut off of GFR 60 mL/min/1.73m^2^ using Grubbs formula for estimation of Cystatin C based eGFR. With values of creatinine as the reference, results were put into 4 categories i.e. True Positive (TP), True Negative (TN), False Positive (FP) and False Negatives (FN). Performance characteristics were calculated sensitivity (TP ÷ (TP + FN) × 100), specificity (TN ÷ (TN + FP) × 100) as indicated in and diagnostic efficiency (TP + TN) ÷ (TP + TN + FN + FP) × 100, Positive likelihood ratio = sensitivity/ (1 − specificity) and negative likelihood ratio = (1 − sensitivity)/specificity. Receiver Operation Curves was drawn to describe the accuracy of concentration levels of Serum Cystatin C based eGFR.

The creatinine clearance was calculated with the C&G formula (1):

1 $${\text{C}}\&\mathrm{G }(140 -\mathrm{ age})\mathrm{ \,x \,weight}/75\mathrm{ \,x\, }{{\text{S}}}_{{\text{cr}}} ({\text{x}}0.85\mathrm{ \,if \,female})$$

Differences between groups for continuous and categorical variables were estimated, respectively, by non-parametric Mann–Whitney U-test for non-normally distributed data or t test for normally distributed data and two proportion tests for categorical variables.

## Results

Of the 914 participants 626 (68.5%) were females and 288 (31.5%) were males. The median age in the study was 38.4 years (IQR 31—45). The Median weight was 61.5 (IQR 52 – 70). The Mean Cystatin C based eGFR (mg/L) concentration was 0.8 mg/L. Of 914 participant 575(63%) were on TDF based regime. The average duration on ART among the participants was 4.9 years. The means and standard deviation of the main blood chemistries were Serum Creatinine 77(31.8), Serum Cystatin C based eGFR 0.8(0.02), Potassium 4.4(0.6), Sodium 140(7.9), and Urea 3.1(1.6). Table 1.

**Table 1:** Demographic and clinical characteristics of the study participants at Mildmay Uganda between 1st Feb-31st Mar2015

	Overall population	Creatinine eGFR	Creatinine eGFR	*p* value
	*n* = 914	< 60 ml/min/1.73m3	> 60 ml/min/1.73m3	
		*n* = 93	*n* = 821	
Gender Female n (%)	626(68.5%)	24(26%)	264(32%)	< 0.01
Male n (%)	288(31.5%)	69(74%)	557(68%)	< 0.01
Age (years), median (IQR)	38.4 (31–45)	48(31–67)	36(18–65)	< 0.01
Weight (Kgs) median (IQR)	61.5(52—70)	50(36–67)	61(36–99)	< 0.01
Duration on ART^a^ (years) median (IQR)	4.9 (0.5–14)	6.6(1.2–12.2)	4.6(0.6–14.2)	0.929
ART regime TDF regime n (%)	575 (62.9%)	55(59%)	520(63%)	0.78
Non TDFn (%)	339 (37.1%)	38(41%)	301(37%)	0.56
Potassium mean (SD)^d^	4.4 (0.55)	4.5(0.56)	4.4(0.54)	< 0.01
Sodium mean (SD)^c^	140 (7.9)	141(7.59)	140(7.99)	< 0.01
Urea mean (SD)^b^	3.1 (1.6)	3.8(1.5)	3.1(1.5)	< 0.01
Cystatin C mean (SD)	0.8 (0.2)	1.01(0.5)	0.7(0.14)	< 0.01

*eGFR* Estimated glomerular filtration rate

^a^19 missing duration on ART

^b^2 missing urea

^c^23 missing Sodium

^d^339 missing Potassium

**Fig. 1:**
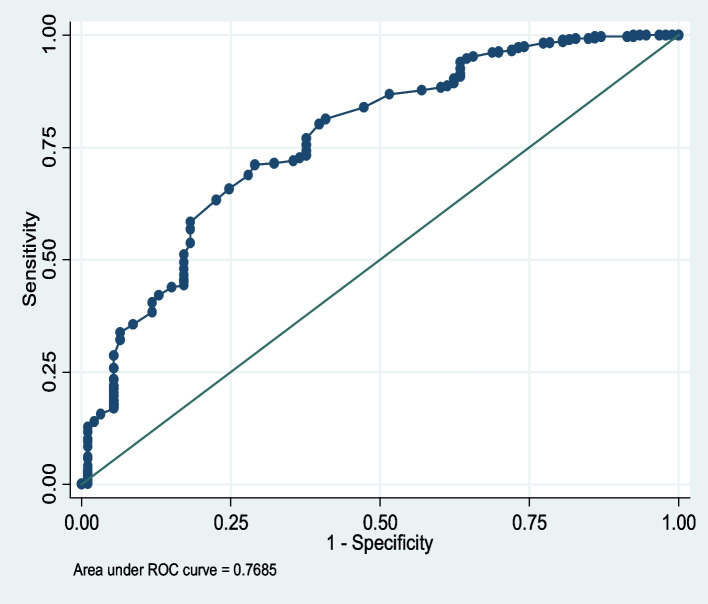
Showing receiver operating characteristics curve analysis of diagnostic accuracy of eGFR Cystatin C based eGFR. The eGFR Creatinine was used as a reference at cut off value 60 mL/min/1.73 m.^2^

The sensitivity of Cystatin C based eGFR was (15.1%) and specificity of (99.3%) with the Positive and negative predictive values of 70% and 91.16% respectively Table 2. The positive and negative likelihood ratios are 21 and 0.8 respectively the prevalence of CKD was 10.2% as shown in Table 3. Diagnostic efficiency of Cystatin C based eGFR at different levels with area under curve was (AUC = 0.77, 95% CI = 0.72–0.82) as shown in Fig. 1.

**Table 2:** 2 × 2 Contingency table showing results of eGFR of Creatinine and eGFR Cystatin C based eGFR at cut off of 60 mL/min/1.73m^3^

		**eGFR Creatinine**		
		< 60 mL/min/1.73m^2^	> 60 mL/min/1.73m^2^	
Serum Cystatin C based eGFR	< 60 mL/min/1.73m^2^	14	6	20
	> 60 mL/min/1.73m^2^	79	815	894
		93	821	914

**Table 3:** Sensitivity, specificity, predictive values and likelihood ratios accuracy of Cystatin C based eGFR in studied subjects categorized at 60 mL/min/1.73m^2^

Performance Characteristics	GFR Cystatin C based eGFR (% and 95 CI)
Sensitivity	15.1 (8.5, 23.9)
Specificity	99.3 (98.4, 99.7)
Positive Predictive values	70.0 (45.7, 88.1)
Negative Predictive values	91.2 (89.1, 92.9)
Positive Likelihood Ratios	21
Negative likelihood Ratios	0.8
Prevalence of CKD	10.20%

## Discussion

In this cross-sectional study, we evaluated the clinical utility of Cystatin C based eGFR in assessing renal function among patients with HIV/AIDS on ART at Mildmay Uganda. We used the Cockcroft-Gault formula as the reference for eGFR estimates. The prevalence of kidney disease in our study population was 10.2%. The sensitivity of Cystatin C based eGFR was (15.1%) and specificity of (99.3%) with the Positive and negative predictive values of 70% and 91.16% respectively. The positive and negative likelihood ratios are 21 and 0.8 respectively. The diagnostic efficiency of Cystatin C based eGFR at different levels with area under curve was (AUC = 0.77, 95% CI = 0.72–0.82).

Our study had strengths and limitations. A major strength being we analyzed serum Cystatin C-based eGFR in a laboratory equipped with a state-of-the-art chemistry analyzer and our adherence to strict good clinical laboratory practice (GCLP) helped minimize systematic error. However, due to technical requirements and safety considerations, we were unable to measure GFR using gold standard methods such as exogenous markers like Inexhol or inulin. Additionally, the absence of published likelihood ratios limited our ability to compare our findings with those from other studies. The study was done in a routine clinical setup that mimics the general situation in many resources limited settings. However, this study was carried out in a clinical center of excellence where most of the services like drug supply, laboratory supplies, expert physicians and consultants are quite reliable which may be different from what is available in majority of the health facilities offering similar services countywide.

Similarly, large bodies of research [31, 32] have documented prevalence of kidney diseases in HIV-infected persons. Jones et al. and Wools-Kaloustian and colleagues reported prevalence of GFR less than 60 mL/min/1.73 m^2^ between 11.5% and 15.2%, respectively [10, 33]. Contrary to our findings, Kalyesubula et al. (2015) reported a prevalence of 14.8% in kidney disease in Uganda [34, 35].

The sensitivity of Cystatin C based eGFR based eGFR was 15.1% and specificity of 99.3% with the positive and negative predictive values of 70% and 91.16% respectively for Cystatin C based eGFR formula at a cut off 60 mL/min/1.73m^2^. The high specificity of Cystatin C based eGFR means the test correctly identifies disease free individuals 99.3% of the times. This result suggests that we can use this test as a confirmatory test for kidney disease. However, the low sensitivity of Cystatin C based eGFR which limits its ability to be used as screening test could have been influenced by choice of the reference method. The high negative predictive value means 91% of patients who have a negative (normal GFR as defined by Cystatin C based eGFR) do not have kidney disease. However, the low positive predictive values 70% in our study could be attributed to the low prevalence of kidney disease among the study participants. Contrary to our findings, a study by Chantrel et al. 2000, reported a sensitivity of Cystatin C based eGFR of 75% [36]. However, this variation could be attributed to the selection of the reference standard, which could have influenced the sensitivity of Cystatin C-based eGFR. Nevertheless, when compared to creatinine, Cystatin C-based eGFR demonstrated a performance more closely aligned with the GFR reference examination. Furthermore, assays for cystatin C using the immunonephelometric procedure showed a stronger correlation. Therefore, reclassifying patients based on Cystatin-based eGFR could offer advantages for both patient care and healthcare systems.

Our finding showed a high positive likelihood ratio of 21(95% CI = 8.1, 55). Converting the likelihood ratios to odds using Bayes’ theorem gave posttest odds of 2.4 with posterior probability 70%, (95% CI 48—86%) given a positive test. This result implies given a positive test (reduced GFR as determined by Cystatin C based eGFR) increases the probability of having renal disease to 70%. The negative likelihood ratio of 0.8, (95% CI = 0.8–0.9) with the posterior probability 9% (95% CI = 8% -10%) and odds of (0.1). This means the chances of having a negative test (normal GFR) as determined by Cystatin C based eGFR in a subject with renal disease is only 9%. These results further imply that Cystatin C based eGFR will correctly identify 91% of individuals without renal disease. Thus, in conjunction with other renal tests we can use Cystatin C based eGFR as a confirmatory test to rule out kidney disease.

In this study we report a reasonable accuracy of Cystatin C based eGFR based formula in classifying kidney dysfunction as indicated by the Area under Curve (AUC) of 0.768, (95% CI = 0.71, 0.821) on the receiver operating characteristics curve (Fig. 1). This result means Cystatin C based eGFR will correctly identify 76.8% have kidney disease (reduced GFR as determined by Cystatin C based eGFR). These findings are in agreement with the study conducted by [37], in assessment of serum Cystatin C based eGFR as an endogenous marker of renal function in patients with mild to moderate impairment of kidney function, the study reported that serum Cystatin C based eGFR is a reliable marker in patients with mildly to moderately impaired kidney function and has a fair diagnostic accuracy than serum Creatinine.

Our study implies that the appropriate use of ART may attenuate the growing epidemic of HIV-1-associated nephropathy. These findings are similar to studies conducted by [38], HIV-1-associated nephropathy and response to highly-active antiretroviral therapy that indicated a substantial reduction in human immunodeficiency virus-1-associated nephropathy incidence chronic kidney disease among patients on ART. Other studies indicated that ART improves renal function by reduction in HIV associated nephropathy and HIV immune complex disease which are directly attributed to HIV infection [39–43]. We found no significant difference in kidney function in HIV positive adults treated with TDF or non-TDF NNRTI based ART regimen (*p* = 0.87) and well as duration on HAART (*p* = 0.93). In line with our study [44], We found that Renal function remained stable with no difference between HIV patients treated with TDF or non-TDF NNRTI based ART regimen.

Nonetheless, our study offers valuable insights into the potential benefits of using Cystatin C-based eGFR in diagnosing and managing renal disease in HIV-positive individuals, particularly highlighting the high precision of Cystatin C-based eGFR formulas. This precision enables easy comparison of eGFR estimates between different laboratories and time points, regardless of the specific formula used.

In resource-limited settings, where access to advanced diagnostics may be limited, using Cystatin C-based eGFR as a confirmatory test could be a practical approach. This could help reduce the need for more expensive or invasive tests and facilitate timely diagnosis and management of kidney disease in HIV/AIDS patients on HAART.

## Conclusion

Cystatin C based eGFR had a high specificity of 99.3% but a low sensitivity of 15.1%, this means that Cystatin C based eGFR can be used as a confirmatory test not as a screening test, a positive and a negative predictive value of 70% and 91.2%, a high positive likelihood ratio of 21 and a negative predictive value 0.8 with a high diagnostic accuracy of Cystatin C based eGFR with area under curve of 0.77.

## Recommendations

In our study, we have demonstrated the utility of Cystatin C-based eGFR as a valuable tool for assessing renal function, particularly in settings where inulin availability is limited. The use of Cystatin C-based eGFR offers a practical and reliable alternative for assessing renal function, especially in resource-limited settings. We recommend that ART programs could explore Cystatin C based eGFR as a confirmatory test for assessment of renal function in HIV&AIDS patients. But also, to be used in conjunction with other blood/urine biochemistries such as serum Creatinine and protein in urine.

While Inflammation can indeed affect Cystatin C levels, leading to inaccuracies in eGFR calculations, we did not measure CRP in this study, and thus future studies could explore the impact of inflammation on Cystatin C-based eGFR results by considering including CRP testing in the study design.

## Data Availability

The datasets used and/or analyzed during the current study are available from the corresponding author on reasonable request.

## References

[1] Isolation of a T-Lymphotropic Retrovirus from a Patient at Risk for Acquired Immune Deficiency Syndrome (AIDS) | Science. Available from: https://www.science.org/doi/10.1126/science.6189183. Cited 2024 Feb 1. 10.1126/science.6189183 6189183

[2] Broder S, Gallo RC (1984). A pathogenic retrovirus (HTLV-III) linked to AIDS. N Engl J Med.

[3] 2013 UNAIDS Report on the global AIDS epidemic | UNAIDS. Available from: https://www.unaids.org/en/resources/documents/2013/20130923_UNAIDS_Global_Report_2013. Cited 2024 Feb 1.

[4] 2023 Report - UNAIDS - Global Report; 2023. Available from: https://thepath.unaids.org/. Cited 2024 Mar 13.

[5] Global, regional, and national life expectancy, all-cause mortality, and cause-specific mortality for 249 causes of death, 1980–2015: a systematic analysis for the Global Burden of Disease Study. 2015. Lancet. Available from: https://www.thelancet.com/journals/lancet/article/PIIS0140-6736(16)31012-1/fulltext. Cited 2024 Feb 1. 10.1016/S0140-6736(16)31012-1 PMC5388903 27733281

[6] 2012 UNAIDS Report on the Global AIDS Epidemic | UNAIDS. Available from: https://www.unaids.org/en/resources/documents/2012/20121120_UNAIDS_Global_Report_2012. Cited 2024 Feb 1.

[7] Replication and compartmentalization of HIV-1 in kidney epithelium of patients with HIV-associated nephropathy - PubMed. Available from: https://pubmed.ncbi.nlm.nih.gov/11984599/. Cited 2024 Feb 1. 10.1038/nm0502-522 11984599

[8] Microcyst formation and HIV-1 gene expression occur in multiple nephron segments in HIV-associated nephropathy - PubMed. Available from: https://pubmed.ncbi.nlm.nih.gov/11729233/. Cited 2024 Feb 1. 10.1681/ASN.V12122645 11729233

[9] Izzedine H, Launay-Vacher V, Deray G (2005). Antiviral Drug-Induced Nephrotoxicity. Am J Kidney Dis.

[10] Jones R, Scott C, Nelson M, Levy J (2007). Renal complications in HIV. Int J Clin Pract.

[11] Guidelines for the management of chronic kidney disease in HIV-infected patients: Recommendations of the HIV Medicine Association of the Infectious Diseases Society of America — Northwestern Scholars. Available from: https://www.scholars.northwestern.edu/en/publications/guidelines-for-the-management-of-chronic-kidney-disease-in-hiv-in. Cited 2024 Feb 1. 10.1086/430257 15889353

[12] Cohen AH, Sun NC, Shapshak P, Imagawa DT (1989). Demonstration of human immunodeficiency virus in renal epithelium in HIV-associated nephropathy. Mod Pathol.

[13] Valeri A, Neusy AJ (1991). Acute and chronic renal disease in hospitalized AIDS patients. Clin Nephrol.

[14] Renal aspects of therapy for human immunodeficiency virus an... J Am Soc Nephrol. Available from: https://journals.lww.com/jasn/abstract/1991/03000/renal_aspects_of_therapy_for_human.4.aspx. Cited 2024 Feb 1. 10.1681/ASN.V191061 1912406

[15] Rao TKS, Friedman EA, Nicastri AD (1987). The types of renal disease in the acquired immunodeficiency syndrome. N Engl J Med.

[16] Liyanage T, Ninomiya T, Jha V, Neal B, Patrice HM, Okpechi I (2015). Worldwide access to treatment for end-stage kidney disease: a systematic review. Lancet.

[17] The Epidemiology of Chronic Kidney Disease in Northern Tanzania: A Population-Based Survey. PLoS One. Available from: https://journals.plos.org/plosone/article?id=10.1371/journal.pone.0124506. Cited 2024 Feb 1. 10.1371/journal.pone.0124506 PMC4401757 25886472

[18] A 4-year survey of the spectrum of renal disease at a National Referral Hospital Outpatient Clinic in Uganda - Kidney International. Available from: https://www.kidney-international.org/article/S0085-2538(15)30181-2/fulltext. Cited 2024 Feb 1. 10.1038/ki.2014.411 25723640

[19] Baumgarten M, Gehr T (2011). Chronic kidney disease: detection and evaluation. Am Fam Physician.

[20] R P, R S, Jk A, A B, J E, Bg F, et al. Long-term monitoring in primary care for chronic kidney disease and chronic heart failure: a multi-method research programme. 2021; Available from: https://www.phc.ox.ac.uk/publications/1193584. Cited 2024 Mar 27. 34469090

[21] Jo JY, Ryu SA, Kim JI, Lee EH, Choi IC (2019). Comparison of five glomerular filtration rate estimating equations as predictors of acute kidney injury after cardiovascular surgery. Sci Rep.

[22] Screening for renal disease using serum creatinine: who are we missing? | Nephrology Dialysis Transplantation | Oxford Academic. Available from: https://academic.oup.com/ndt/article/16/5/1042/1807743. Cited 2024 Feb 1. 10.1093/ndt/16.5.1042 11328914

[23] Serum Creatinine Is an Inadequate Screening Test for Renal Failure in Elderly Patients | Nephrology | JAMA Internal Medicine | JAMA Network. Available from: https://jamanetwork.com/journals/jamainternalmedicine/fullarticle/215075. Cited 2024 Feb 1. 10.1001/archinte.163.3.356 12578517

[24] Prediction of Creatinine Clearance from Serum Creatinine | Nephron | Karger Publishers. Available from: https://karger.com/nef/article-abstract/16/1/31/212046/Prediction-of-Creatinine-Clearance-from-Serum?redirectedFrom=fulltext. Cited 2024 Feb 1.

[25] Grubb A, Björk J, Lindström V, Sterner G, Bondesson P, Nyman U (2005). A cystatin C-based formula without anthropometric variables estimates glomerular filtration rate better than creatinine clearance using the Cockcroft-Gault formula. Scand J Clin Lab Invest.

[26] Filler G, Bökenkamp A, Hofmann W, Le Bricon T, Martínez-Brú C, Grubb A (2005). Cystatin C as a marker of GFR—history, indications, and future research. Clin Biochem.

[27] Donadio C, Lucchesi A, Ardini M, Giordani R, Cystatin C (2001). β2-microglobulin, and retinol-binding protein as indicators of glomerular filtration rate: comparison with plasma creatinine. J Pharm Biomed Anal.

[28] Reed CH (2000). Diagnostic applications of cystatin C. Br J Biomed Sci.

[29] Laterza OF, Price CP, Scott MG (2002). Cystatin C: an improved estimator of glomerular filtration rate?. Clin Chem.

[30] Horio M, Orita Y, Manabe S, Sakata M, Fukunaga M (1997). Formula and nomogram for predicting creatinine clearance from serum creatinine concentration. Clin Exp Nephrol..

[31] Chaparro AI, Mitchell CD, Abitbol CL, Wilkinson JD, Baldarrago G, Lopez E (2008). Proteinuria in children infected with the human immunodeficiency virus. J Pediatr.

[32] Eke FU, Anochie IC, Okpere AN (2007). Proteinuria in HIV positive children a pilot study. Pediatr Nephrol.

[33] Wools-Kaloustian KK, Gupta SK (2008). Will there be an epidemic of HIV-related chronic kidney disease in sub-Saharan Africa? Too soon to tell. Kidney Int.

[34] Troyanov S, Cattran D, Coppo R (2015). The authors reply. Kidney Int.

[35] Kalyesubula R, Lunyera J, Makanga G, Kirenga B, Amukele TK (2015). A 4-year survey of the spectrum of renal disease at a National Referral Hospital Outpatient Clinic in Uganda. Kidney Int.

[36] Chantrel F, Agin A, Offner M, Koehl C, Moulin B, Hannedouche T (2000). Comparison of cystatin C versus creatinine for detection of mild renal failure. Clin Nephrol.

[37] Hojs R, Bevc S, Ekart R, Gorenjak M, Puklavec L (2008). Serum Cystatin C as an endogenous marker of renal function in patients with chronic kidney disease. Ren Fail.

[38] Wali RK, Drachenberg CI, Papadimitriou JC, Keay S, Ramos E (1998). HIV-1-associated nephropathy and response to highly-active antiretroviral therapy. The Lancet.

[39] Leventhal JS, Ross MJ (2008). Pathogenesis of HIV-Associated Nephropathy. Semin Nephrol.

[40] Eneyew K, Seifu D, Amogne W, Menon MKC (2016). Assessment of Renal Function among HIV-Infected Patients on Combination Antiretroviral Therapy at Tikur Anbessa Specialized Hospital, Addis Ababa. Ethiopia Technol Invest.

[41] Marras D, Bruggeman LA, Gao F, Tanji N, Mansukhani MM, Cara A (2002). Replication and compartmentalization of HIV-1 in kidney epithelium of patients with HIV-associated nephropathy. Nat Med.

[42] Bruggeman LA, Ross MD, Tanji N, Cara A, Dikman S, Gordon RE (2000). Renal Epithelium Is a Previously Unrecognized Site of HIV-1 Infection. J Am Soc Nephrol.

[43] Ross MJ, Klotman PE (2004). HIV-associated nephropathy. AIDS.

[44] Yilma D, Abdissa A, Kæstel P, Tesfaye M, Olsen MF, Girma T (2020). Renal function in Ethiopian HIV-positive adults on antiretroviral treatment with and without tenofovir. BMC Infect Dis.

